# A Wind Estimation Method with an Unmanned Rotorcraft for Environmental Monitoring Tasks

**DOI:** 10.3390/s18124504

**Published:** 2018-12-19

**Authors:** Jia-Ying Wang, Bing Luo, Ming Zeng, Qing-Hao Meng

**Affiliations:** 1 Institute of Robotics and Autonomous Systems, Tianjin Key Laboratory of Process Measurement and Control, School of Electrical and Information Engineering, Tianjin University, Tianjin 300072, China; wjy0709@tju.edu.cn (J.-Y.W.); zengming@tju.edu.cn (M.Z.); 2 National Computer Network Emergency Response Technical Team/Coordination Center of China, Beijing 100029, China; luobing@cert.org.cn

**Keywords:** environmental monitoring, unmanned aerial vehicle, wind estimation, extended state observer

## Abstract

Wind velocity (strength and direction) is an important parameter for unmanned aerial vehicle (UAV)-based environmental monitoring tasks. A novel wind velocity estimation method is proposed for rotorcrafts. Based on an extended state observer, this method derives the wind disturbance from rotors’ speeds and rotorcraft’s acceleration and position. Then the wind disturbance is scaled to calculate the airspeed vector, which is substituted into a wind triangle to obtain the wind velocity. Easy-to-implement methods for calculating the rotorcraft’s thrust and drag coefficient are also proposed, which are important parameters to obtain the wind drag and the airspeed, respectively. Simulations and experiments using a quadrotor in both hovering and flight conditions have validated the proposed method.

## 1. Introduction

With advantages of hovering capability and high maneuverability, unmanned rotorcrafts (URs) have become popular in a diverse range of environmental monitoring applications, such as atmospheric measurement [1] and air pollution tracing [2,3]. In environmental monitoring activities, the wind profile plays a significant role, not only because UR flight performance is vulnerable to unpredictable wind conditions, but also because it is a kind of important information. For example, wind information is usually required in typical gas-tracing approaches such as anemotaxis algorithms [4,5], fluid-engineering-based methods [6,7] and statistical methods [8,9,10]. As a consequence, it is valuable for engineering practice that URs be designed with the ability to measure wind strength/direction.

Mounting wind sensors, such as anemometers [11] and pitot probes [12], on URs may be a straightforward way to solve the problem, however, anemometers and other auxiliary sensors are typically bulky and heavy in contrast to the valuable payload of the UR. For saving payload and space for other sensors (for instance, gas sensors) needed for the environment monitoring tasks, components that add extra weight should be avoided. Although pitot tubes are usually small, their measurement accuracy tends to be low when URs fly at low speed. Moreover, because the turbulence induced by the rotors would strongly deteriorate the sensor outputs, this direct methodology cannot yield reliable results on URs.

The ideal practical solution should possess four features: (1) not be susceptible to rotor wake influence; (2) not require additional hardware to save valuable space for other sensors; (3) whether the UR is hovering or flying, the wind data can be measured accurately; (4) in order to save time costs for subsequent algorithm development, the method should be applicable to the broadest types of URs and easy to operate.

This paper proposes a wind estimation method for URs with the four features mentioned above. The method only requires the inertial measurement unit (IMU) outputs, rotor speeds and the rotorcraft’s position information to work properly under hovering and flight conditions. In addition, we also propose easy-to-implement methods to calculate the rotorcraft’s thrust and drag coefficient in the wind estimation algorithm.

The remainder of the paper is organized as follows: related work is firstly described in Section 2. Section 3 presents the wind estimation method, including the dynamic model of the rotorcraft, and the thrust, wind drag and drag coefficient of the rotorcraft calculation methods. Section 4 and Section 5 describe the simulation and experimental results, respectively. Finally, conclusions are given in Section 6.

## 2. Related Works

Previous works can be divided into two categories: theoretical methods and experimental approaches. Waslander and Wang [13] developed exquisite models for quadrotor aerodynamics and proposed a memoryless estimation method on the basis of these models. Gonzalez-Rocha et al. [14] proposed a wind estimation method based on motion models and identified the parameters of the motion models using a wind tunnel and flight data. The same kind of methods were also adopted by Muller [15,16], Sikkel [17] and Schiano [18]. Based on the kinematics mechanism of the quadrotor, these methods establish complex motion models that are independent of experimental conditions. However, it is usually difficult to determine the accurate models and parameters that can describe the interaction between the wind and the rotorcraft exactly.

Neumann et al. [19], Palomaki et al. [20], Eu et al. [21] and Marino et al. [1] bypassed the theoretical descriptions and directly established the mapping from the quadrotor’s inclination angle with/without the power consumption to the wind strength/direction. Instead of modeling the complex relationship between the wind and the quadrotor, these experimental approaches mine the wind information from the motion data of the quadrotor, but the prerequisite is that a wind tunnel is required to obtain training data. Besides, these methods obtain the relationship between the wind velocity and the motion data in hovering conditions. However, when the quadrotor is in flight conditions, the inclination angle of the quadrotor not only contains the passive inclination caused by the air drag, but also includes the active inclination for flying along the target path. It is relatively difficult to separate the inclination angle caused by wind from that caused by motors. As a consequence, even though some methods use a positioning system, e.g., global position system (GPS), to enable wind estimation during flight, most approaches may not provide estimation results with high precision.

Song et al. [22] calculated the thrust of a quadrotor based on blade element theory to estimate the wind speed. This method took the rotors rather than the frame as an object to study the elaborate relationship between the wind resistance and the wind speed. However, the wind direction was also estimated by the inclination-angle-measurement method. In addition, a self-designed special experimental device called hardware-in-the-loop simulation (HILS) system was required for thrust measurement, parameter calibration and validation experiments. Hüllmann et al. [23] analyzed the transfer function describing the relationship between pulse width modulation (PWM) duty cycle and motor speed, which may be a way to calculate the thrust.

In this paper, we propose a novel wind estimation method for URs, which takes the flight acceleration into account. Inspired by the studies of active disturbance rejection control (ADRC) [24,25], the proposed method considers the wind as an acceleration disturbance of the rotorcraft and employs an extended state observer (ESO) [26,27] to estimate this wind disturbance from rotors’ speeds and rotorcraft’s acceleration and position during flight. Then the wind disturbance is used to calculate the airspeed of the quadrotor, and finally the wind velocity can be obtained.

## 3. Wind Velocity Estimation Method

### 3.1. Dynamic Model of the Rotorcraft

**Figure 1:**
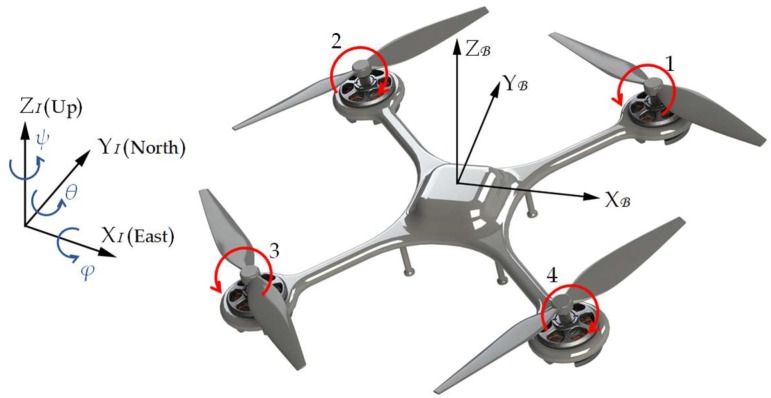
The inertia frame and the body frame of the rotorcraft. The three axes of the body frame B and inertia frame I are illustrated by the black vectors. The blue arrows and letters represent Euler angles: roll (*φ*), pitch (*θ*), and yaw (*ψ*). The red arrows show the rotational directions of the rotors.

The inertial frame I and body frame B are commonly used to describe the motion of aircrafts. Two frames can be converted to each other through Euler angles: roll (*φ*), pitch (*θ*), and yaw (*ψ*). Taking a quadrotor as an instance, the inertial frame I and body frame B are shown in Figure 1. 

In this figure, the origin of the body frame is located at the geometric center of the rotorcraft, and the midpoint of the line segment connecting rotors 1 and 2 is defined as the front of the rotorcraft, which is also the positive direction of Y-axis in the body frame (Y_B_). The Euler angles are defined in the inertia frame, and the yaw angle *ψ* is zero when the front side of the quadrotor just points to North (Y_I_). The rotation matrix from I to B is calculated using the Euler angles as:

(1) $$R_{I}^{B}=\left[\begin{array}{ccc} \cos\psi \cos\theta & \sin\psi \cos\theta & -\sin\theta \\ \sin\varphi \cos\psi \sin\theta -\cos\varphi \sin\psi & \sin\varphi \sin\psi \sin\theta +\cos\varphi \cos\psi & \sin\varphi \cos\theta \\ \cos\varphi \cos\psi \sin\theta +\sin\varphi \sin\psi & \cos\varphi \sin\psi \sin\theta -\sin\varphi \cos\psi & \cos\varphi \cos\theta \end{array}\right]$$

The rotation matrix from B to I is defined by transposing $R_{I}^{B}$, that is $R_{B}^{I}\equiv R_{I}^{B^{-1}}\equiv R_{I}^{B^{T}}$.

**Figure 2:**
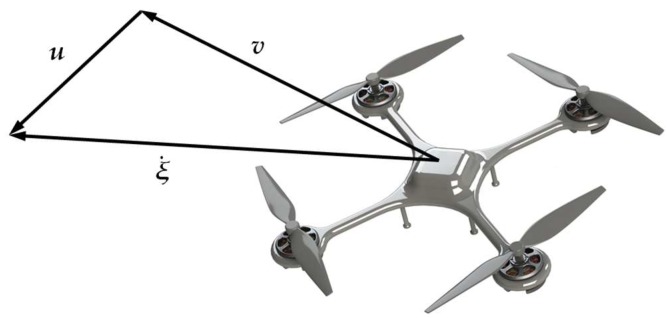
The relationship of the three velocities. $\dot{\xi }$ is the ground velocity, ***v*** is the airspeed vector and ***u*** is the wind velocity.

The well-known wind triangle (see Figure 2) describes the relationship of the three velocities of the rotorcraft, i.e., ground velocity $\dot{\xi }$ (***ξ*** is the position), airspeed ***v*** (the flight velocity relative to the air) and wind velocity ***u***. 

According to the force analysis, the resultant force of the rotorcraft is generated by its gravity, the total thrust and the wind drag, which can be expressed in *I* as:

(2) $$m\ddot{\xi }=G+R_{B}^{I}T_{B}+R_{B}^{I}F_{B}$$

where *m* is the mass of the rotorcraft and $\ddot{\xi }$ is the acceleration. The gravity of the rotorcraft in I is defined as $G=m{\left[\begin{array}{ccc} 0 & 0 & -g \end{array}\right]}^{T}$. ***T***_B_ and ***F***_B_ are the total thrust and the wind drag in B, respectively. On the basis of the momentum theory and the conservation of energy, the thrust produced by a rotor is proportional to the square of its rotation speed. Thus, the total thrust of the rotorcraft in B is described as:

(3) $$T_{B}={\left[\begin{array}{ccc} 0 & 0 & k\sum_{i=1}^{N}\Omega_{i}^{2} \end{array}\right]}^{T}$$

where *k* is the thrust coefficient, *N* is the number of the rotors, and the rotation speed of each rotor is denoted as Ω*_i_* (*i* = 1, 2, 3, …, *N*). The acceleration of the rotorcraft generated by the total thrust in *I* is denoted as $a_{T}$, which can be written as:

(4) $$a_{T}=\frac{1}{m}R_{B}^{I}T_{B}=\frac{1}{m}R_{B}^{I}{\left[\begin{array}{ccc} 0 & 0 & k\sum_{i=1}^{N}\Omega_{i}^{2} \end{array}\right]}^{T}$$

In consideration of the limited flight speed of the rotorcraft, the wind drag caused by the relative movement between the rotorcraft and the air can be modeled referring to [28,29] as:

(5) $$F_{B}=-\;R_{I}^{B}v\circ C$$

where ***v*** = (*v_x_*, *v_y_*, *v_z_*)^T^ is the airspeed vector of the rotorcraft. ***C*** = (*c_x_*, *c_y_*, *c_z_*)^T^ is the drag coefficient which associates the linear velocities to the drag force. The operator ∘ refers to the Hadamard product. The acceleration of the rotorcraft generated by the wind drag in *I* is denoted by $a_{F}$, which can be expressed as:

(6) $$a_{F}=\frac{1}{m}R_{B}^{I}F_{B}=-\frac{1}{m}R_{B}^{I}(R_{I}^{B}v\;\circ C)$$

After the acceleration $\ddot{\xi }$ and the thrust ***T***_B_ have been measured, the wind-drag acceleration can be obtained by:

(7) $$a_{F}=\ddot{\xi }-{\left[\begin{array}{ccc} 0 & 0 & -g \end{array}\right]}^{T}-a_{T}$$

Then, from Equation (7), the airspeed of the rotorcraft can be deduced as:

(8) $$v=-ma_{F}\circ {\left[\begin{array}{ccc} \frac{1}{c_{x}} & \frac{1}{c_{y}} & \frac{1}{c_{z}} \end{array}\right]}^{T}$$

Finally, the wind velocity can be obtained according to the wind triangle:

(9) $$u=\dot{\xi }-v$$

**Figure 3:**
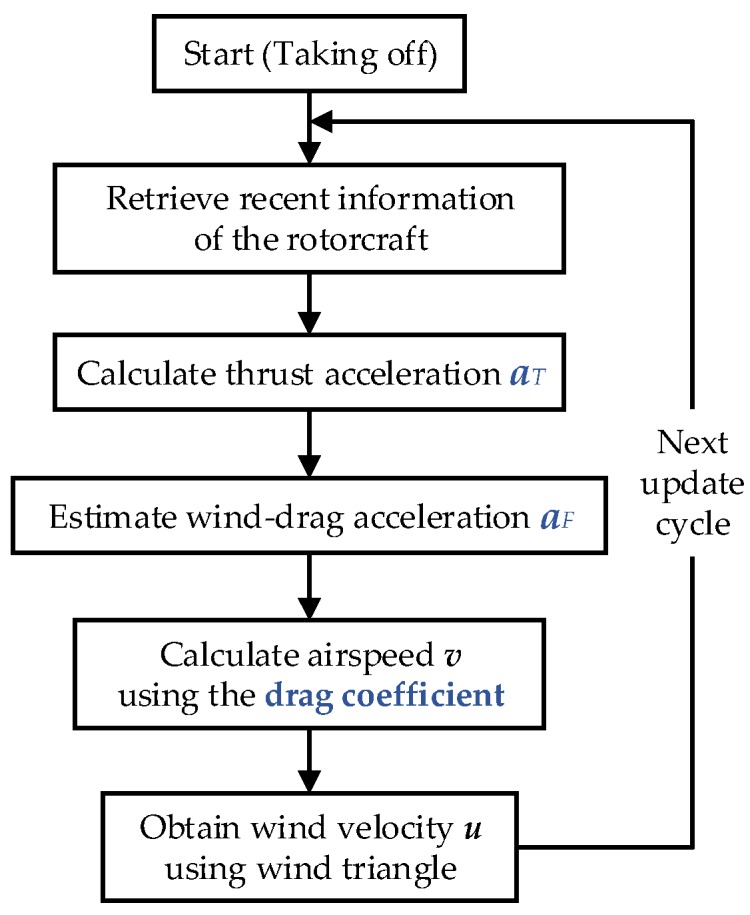
Scheme of the wind estimation method.

In summary, the scheme of the wind estimation method is presented in Figure 3.

In order to estimate the wind velocity, there are three essential factors to be solved:

(1) the thrust acceleration $a_{T}$, which is the key to calculate $a_{F}$;
(2) the wind-drag acceleration $a_{F}$. The noise caused by rotorcraft’s accelerometer (for measuring $\ddot{\xi }$) and dynamic changes of motors’ supplied voltages (for calculating $a_{T}$) will deteriorate the computed result of $a_{F}$ by Equation (8) using instantaneous measurements of $\ddot{\xi }$ and $a_{T}$. Compared to direct calculation by Equation (8), calculating $a_{F}$ by a more efficient method is a crucial step;
(3) the drag coefficient ***C***, which is an important parameter to obtain ***v***.

The calculation methods of $a_{T}$, $a_{F}$ and ***C*** are briefly described in Section 3.2, Section 3.3 and Section 3.4, respectively.

### 3.2. Calculation of the Thrust Acceleration

The traditional method to obtain the value of $a_{T}$ is by conducting experiments to measure thrusts at different rotor speeds and then calculating the thrust coefficient *k*. However, the traditional method involves arduous workload because of two significant barriers: (1) simultaneous measurement of the rotor speed and the thrust usually requires a special experimental device, for instance, a hardware-in-the-loop simulation (HILS) system in [22]; (2) when the rotor’s shape changes, *k* should be remeasured. 

In order to make the wind estimation method manageable and broadly applicable for multiple types of rotorcrafts, we firstly design a simple method to measure the value of $a_{T}$**,** which bypasses the design of special devices and the measurement of *k*.

When the rotorcraft is hovering stably ($\ddot{\xi }{\;=\;0,\;v\;=\;0,\;a}_{F}\;=\;0$) in a windless environment (u = 0), the total thrust equals to the gravity, which can be expanded as:

(10) $$R_{B}^{I}{\left[\begin{array}{ccc} 0 & 0 & k\sum_{i=1}^{N}\Omega_{i0}^{2} \end{array}\right]}^{T}=m{\left[\begin{array}{ccc} 0 & 0 & g \end{array}\right]}^{T}$$

where $\Omega_{i0}$ denotes the speed of the *i*th rotor when the rotorcraft maintains a stable hover in the windless environment. At this moment, φ and *θ* both equal to zero, so Equation (11) can be simplified as:

(11) $$\frac{k}{m}\sum_{i=1}^{N}\Omega_{i0}^{2}=g$$

It is generally known that most of the rotorcrafts use motors as power units. Motor’s speed $\Omega_{i}$ approximately depends linearly on the input voltage or the equivalent voltage of the PWM input signal, that is:

(12) $$\Omega_{i}=k_{p}U_{i}$$

where *k_p_* is a scaling factor, and *U_i_* is the (equivalent) voltage value of the *i*th rotor. Thus, Equation (12) can be rewritten as:

(13) $$\frac{k\cdot k_{p}^{2}}{m}\sum_{i=1}^{N}U_{i0}^{2}=g$$

where *U_i_*_0_ denotes the (equivalent) voltage of the *i*th rotor motor when the rotorcraft maintains a stable hover in the windless environment. With Equations (13) and (14) introduced, Equation (5) can be expressed as:

(14) $$a_{T}=R_{B}^{I}{\left[\begin{array}{ccc} 0 & 0 & \frac{\sum_{i=1}^{N}U_{i}^{2}}{\sum_{i=1}^{N}U_{i0}^{2}}g \end{array}\right]}^{T}$$

As can be seen in Equation (15), rather than designing a special measuring device to acquire the thrust coefficient *k*, this method only needs to adjust the rotorcraft manually to hovering state in a windless environment (such as an indoor environment with doors and windows closed), and collect the (equivalent) voltages of rotor motors for a period of time.

### 3.3. Estimation of the Wind-Drag Acceleration

If $a_{F}$ is computed directly by Equation (8) using instantaneous measurements of $\ddot{\xi }$ and $a_{T}$, the calculation result will contain much noise caused by rotorcraft’s accelerometer (for measuring $\ddot{\xi }$) and dynamic changes of motors’ PWM signals (for calculating $a_{T}$). Therefore, it is necessary to add a filtering process to obtain the value of $a_{F}$. The equation for calculating $a_{F}$ in (8) can be written as:

(15) $$\ddot{\xi }={\left[\begin{array}{ccc} 0 & 0 & -g \end{array}\right]}^{T}+a_{T}+a_{F}$$

In this equation, the wind-drag acceleration $a_{F}$ can be actually regarded as a disturbance term to the expected acceleration which should have been generated by the resultant force of the total thrust and gravity. This interference, i.e., the wind drag, will further affect the position and flight speed of the rotorcraft by time integration. From this point of view, $a_{F}$ can be measured by an extended state observer (ESO) which can estimate the internal uncertainty and external disturbances of the system [30,31]. If $a_{F}$ is considered as an extended state of the rotorcraft, an ESO is proposed to estimate the state vector ${\left[\begin{array}{ccc} \xi & \dot{\xi } & a_{F} \end{array}\right]}^{T}$. The linear extended state observer (LESO) [32] used in this method is mathematically described as:

(16) $$\begin{array}{l} {\dot{z}}_{1}(t)=z_{2}(t)+3\lambda [\xi (t)-\tilde{\xi }(t)]\\ {\dot{z}}_{2}(t)=z_{3}(t)+3\lambda^{2}[\xi (t)-\tilde{\xi }(t)]+\kappa (t)\\ {\dot{z}}_{3}(t)=\lambda^{3}[\xi (t)-\tilde{\xi }(t)]\\ \tilde{\xi }(t)=z_{1}(t) \end{array}$$

(17) $$\kappa ={\left[\begin{array}{ccc} 0 & 0 & -g \end{array}\right]}^{T}+R_{B}^{I}{\left[\begin{array}{ccc} 0 & 0 & \frac{\sum_{i=1}^{N}U_{i}^{2}}{\sum_{i=1}^{N}U_{i0}^{2}}g \end{array}\right]}^{T}$$

where $z_{1}=\tilde{\xi }$, $z_{2}=\;\tilde{\dot{\xi }}$, $z_{3}{=\tilde{a}}_{F}$ are the estimated values of the position, ground velocity and wind-drag acceleration of the rotorcraft, respectively. λ is an adjustable scalar factor and κ is the acceleration excitation term. Optimization of *λ* refers to maximizing the value of *λ*, subject to the condition that the sensitivity to sensor noises and the delay in sampling are acceptable [32]. The value of *λ* only needs to be coarsely adjusted, and the wind estimation method is not overly sensitive to the precise value.

### 3.4. Calculation of the Drag Coefficient

The drag coefficient can generally either be measured by wind tunnel experiments or be roughly calculated by the windward area of the rotorcraft. In this paper, an easy-to-implement estimation method for the drag coefficient is proposed. In a windless environment ($v=\dot{\xi }$), combined with (9), the drag coefficient can be expressed as:

(18) $$C=-ma_{F}\circ {\left[\begin{array}{ccc} \frac{1}{{\dot{\xi }}_{x}} & \frac{1}{{\dot{\xi }}_{y}} & \frac{1}{{\dot{\xi }}_{z}} \end{array}\right]}^{T}$$

It can be seen that the drag coefficient can be obtained from the estimated value $a_{F}$ and the rotorcraft speed $\dot{\xi }$. The calculation process of estimating the drag coefficient is divided into two steps:

(1) *Calculation of c_x_ and c_y_*. In a windless environment, the rotorcraft is controlled to make back-and-forth movements along a straight line in the horizontal plane. In addition, the yaw angle is changed at each round trip. The flight path can be traced by a waypoint-update mode, which is described as:

(19) $$\begin{array}{l} \xi^{\prime }={\left[\begin{array}{ccc} \xi_{x0}^{\prime } & \xi_{y0}^{\prime }+{\left(-1\right)}^{i}d & \xi_{z0}^{\prime } \end{array}\right]}^{T}\\ \psi^{\prime }=\psi_{0}^{\prime }+i\Delta \psi^{\prime }\\ i=0,\;1,\;2,\;3,\ldots \end{array}$$

where $\xi^{\prime }$ denotes the waypoint (reference value of position), *d* is the distance of the back-and-forth movement, $\psi^{\prime }$ is the reference value of the yaw angle, and $\psi_{0}^{\prime }$ is the initial yaw angle. $\psi^{\prime }$ is updated by a constant angle $\Delta \psi^{\prime }$ with a certain frequency. During the flight of the rotorcraft, $a_{F}$ and $\dot{\xi }$ are acquired in real time. After the data have been collected for a period of time, the time series of the drag coefficient is calculated by (19) using the data when the rotorcraft is flying past the waypoint ${\left(\xi_{x0}^{\prime },\xi_{y0}^{\prime },\xi_{z0}^{\prime }\right)}^{T}$. Then the average value $\bar{c_{x}}$ and $\bar{c_{y}}$ of the series of *c_x_* and *c_y_* are taken as the estimators of *c_x_* and *c_y_*, respectively.

(2) *Calculation of c_z_*. In a windless environment, the rotorcraft is controlled to make vertical jumps and the flight path can be described by a waypoint-update mode, that is:

(20) $$\begin{array}{l} \xi^{\prime }={\left[\begin{array}{ccc} \xi_{x0}^{\prime } & \xi_{y0}^{\prime } & \xi_{z0}^{\prime }+{\left(-1\right)}^{i}h \end{array}\right]}^{T}\\ i=0,\;1,\;2,\;3,\ldots \end{array}$$

where *h* is the magnitude of the vertical jump movement, which flips the symbol with a certain low frequency, resulting in constantly changing hover height of the rotorcraft. During the jump of the rotorcraft, after $a_{F}$ and $\dot{\xi }$ have been collected for a period of time, the time series of the drag coefficient *c_z_* is calculated by (19) using the data when the rotorcraft is flying past the waypoint ${\left(\xi_{x0}^{\prime },\xi_{y0}^{\prime },\xi_{z0}^{\prime }\right)}^{T}$. Finally, the estimated value $\bar{c_{z}}$ is obtained by averaging the series of *c_z_*.

It should be noted that the data near the waypoint ${\left(\xi_{x0}^{\prime },\xi_{y0}^{\prime },\xi_{z0}^{\prime }\right)}^{T}$ are selected as the sample to estimate drag coefficients in both two steps, actually because of the relatively stable flight status in which acceleration and attitude will not fluctuate significantly at this point.

In summary, the computational procedure of the ESO based wind estimation method is presented in Algorithm 1.

**Algorithm 1:** Computational procedure of the wind estimation method**Input: *η*** = (φ, θ, ψ)^T^, ξ, $U=\{U_{i}|i=1,2,3,\cdots ,N\}$, $\sum_{i=1}^{N}U_{i0}^{2}$, λ, *c_x_*, *c_y_*, *c_z_*, Δt, *t_end_*
**Output: *u***
1:
$[z_{1},z_{2},z_{3}]\leftarrow 0$
% Initialize the state vector of the ESO2:**while***t* < *t_end_*
**do**
3:  update ***η***(*t*), ξ(t), ***U***(*t*)
4:  calculate $R_{I}^{B}(\eta )$ and $R_{B}^{I}(\eta )$ using (1) and (2)
5:  calculate κ using (18)
6:  $\varepsilon \leftarrow \xi -z_{1}$
7:  $z_{1}\leftarrow z_{1}+\Delta t(z_{2}+3\lambda \varepsilon )$% Update $\tilde{\xi }$
8:  $z_{2}\leftarrow z_{2}+\Delta t(z_{3}+3\lambda^{2}\varepsilon +\kappa )$% Update $\tilde{\dot{\xi }}$
9:  $z_{3}\leftarrow z_{3}+\Delta t(3\lambda^{3}\varepsilon )$% Update ${\tilde{a}}_{F}$
10:  $a_{F}\leftarrow z_{3}$
11:  $v=-ma_{F}\circ {\left[\begin{array}{ccc} \frac{1}{c_{x}} & \frac{1}{c_{y}} & \frac{1}{c_{z}} \end{array}\right]}^{T}$% Calculate the airspeed of the rotorcraft12:  $u\leftarrow \dot{\xi }-v$% Calculate the wind velocity13:
**end while**



## 4. Simulations and Results

Taking the most commonly used quadrotor as an instance, simulation tests were designed to verify the accuracy and repeatability of the proposed wind estimation method. This paper has written a simulation environment based on the robot active olfaction system (RAOS) [33] and designed several scenarios to compare the inclination-angle-measurement method (inclination method for short) proposed in [19] and this method. The simulation environment consists of the models of the quadrotor and the environmental wind.

### 4.1. Simulation Environment

#### 4.1.1. Model of the Quadrotor

In addition to the dynamic model mentioned in Section 3.1, the attitude model is also essential for modeling the quadrotor. When there is diversity between the four rotor speeds of the quadrotor, the difference between thrusts will change its attitude. The torque of the quadrotor $\tau_{B}={\left(\tau_{\varphi },\tau_{\theta },\tau_{\psi }\right)}^{T}$ in B is expressed as:

(21) $$\tau_{B}=\left[\begin{array}{c} Lk(\Omega_{1}^{2}+\Omega_{2}^{2}-\Omega_{3}^{2}-\Omega_{4}^{2})\\ Lk(\Omega_{2}^{2}+\Omega_{3}^{2}-\Omega_{1}^{2}-\Omega_{4}^{2})\\ b(\Omega_{1}^{2}-\Omega_{2}^{2}+\Omega_{3}^{2}-\Omega_{4}^{2}) \end{array}\right]$$

where *L* is the distance from rotor motor to the barycenter of the quadrotor, *b* is the torque coefficient, and Ω*_i_* (*i* = 1, 2, 3, 4) is the rotation speed of each rotor. The arrangement of the rotors and their rotational directions are depicted in Figure 1.

The relation between the angular velocity ***ω****_B_* in B and the Euler angle vector ***η*** = (φ, θ, ψ)^T^ in I is described as:

(22) $$\omega_{B}=W_{I}^{B}\dot{\eta }$$

where ***W*** is the rotation matrix between the angular velocity and the first derivative of the Euler angle, expressed as:

(23) $$W_{I}^{B}=\left[\begin{array}{ccc} 1 & 0 & -\sin\theta \\ 0 & \cos\varphi & \sin\varphi \cos\theta \\ 0 & -\sin\varphi & \cos\varphi \cos\theta \end{array}\right]$$

(24) $$W_{B}^{I}=\left[\begin{array}{ccc} 1 & \sin\varphi \tan\theta & \cos\varphi \tan\theta \\ 0 & \cos\varphi & -\sin\varphi \\ 0 & \sin\varphi /\cos\theta & \cos\varphi /\cos\theta \end{array}\right]$$

The attitude dynamics equation of the quadrotor (Euler equation) is written in I as:

(25) $$J{\dot{\omega }}_{B}+\omega_{B}\times (J\omega_{B})=\tau_{B}$$

where ***J*** is the rotational inertia of the quadrotor.

#### 4.1.2. Model of Environmental Wind

**Figure 4:**
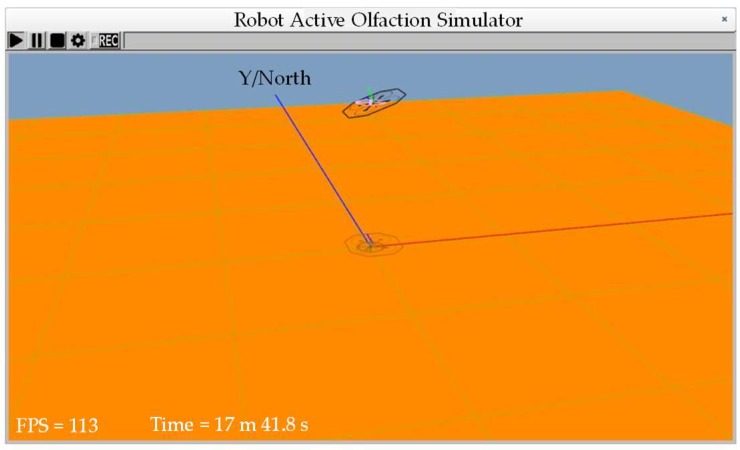
Simulation environment.

The environmental wind is simulated using the colored noise method mentioned in [34], expressed as:

(26) $$u_{w}=u_{0}+u^{\prime }$$

where $u_{0}$ and $u^{\prime }$ are the steady component and the fluctuating component of the wind, respectively. The kinetic model of $u^{\prime }$ can be written by:

(27) $$\frac{d^{2}}{dt^{2}}u_{i}^{\prime }+2\mu_{i}\varsigma_{i}\frac{d}{dt}u_{i}^{\prime }+\varsigma_{i}^{2}u_{i}^{\prime }=G_{i}\varsigma_{i}^{2}\delta_{i},\;i\in \{x,y,z\}$$

where μ is the damping factor, ζ is the bandwidth coefficient, *G* is the gain coefficient, and δ is the white noise. Figure 4 shows the simulation environment where the wind blows eastward (the right-hand side of the figure) and the quadrotor generates an inclination angle in a steady wind to resist the wind disturbance.

### 4.2. Simulation Setup

The reference values for the parameters in the model of the quadrotor are listed in Table 1.

**Figure 5:**
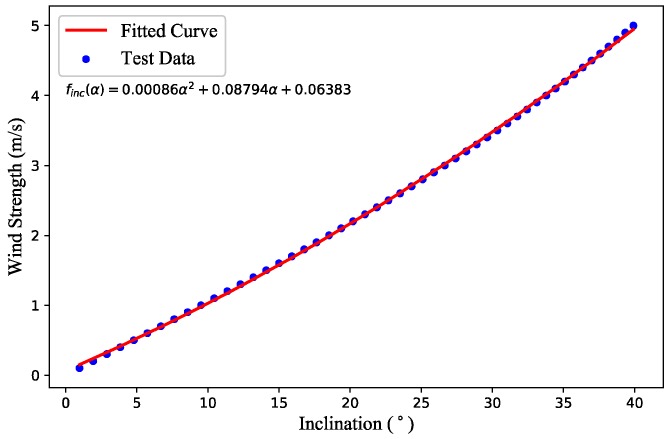
Fitting results of the inclination angles and wind strengths.

As a comparison, the inclination method is also simulated. The relationship between the inclination angle and the wind strength is acquired in the simulation environment. By simulating the wind tunnel experiment, a series of steady winds of different velocities are generated, and then wind strengths as well as inclination angles are recorded when the quadrotor is hovering stably. The fitting results are presented in Figure 5. 

In the proposed method, *λ* is set to 18 which is obtained by several tests according to the parameter adjustment method in [32]. The supplied voltage is 3.7 V in the windless environment, and the quadratic sum of motors’ equivalent voltages is $\sum_{i=1}^{4}U_{i0}^{2}=22.059$ V^2^. The drag coefficient obtained by the method in Section 3.4 is $\left[\begin{array}{ccc} 0.20001 & 0.20001 & 0.830005 \end{array}\right]$, where *d* is set to 25 m and *h* is set to 10 m.

**Figure 6:**
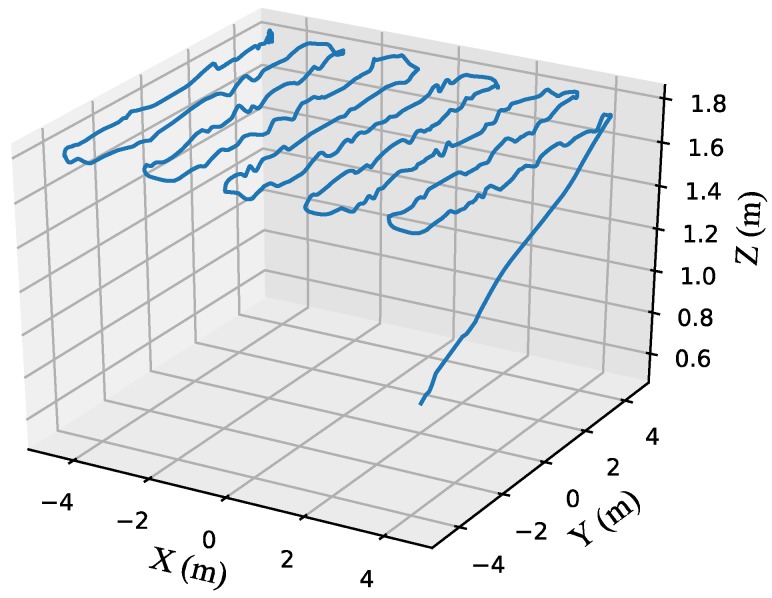
Real flight path of the quadrotor in the time-varying wind field.

Three tests were carried out to investigate the accuracy and repeatability of the proposed method in the simulation:

(1) Test 1: Wind gust estimation with a quadrotor in hovering conditions. The gust wind is simulated with a square wave signal of a 20 s period, and the wind strength is (1, 0, 0) m/s, i.e., the wind blows towards the east. The quadrotor is in hovering condition. The frequencies of the actual and the estimated wind strength/direction signals are set at 50 Hz.
(2) Test 2: Time-varying wind estimation with a quadrotor in hovering conditions. The constant component of the wind strength is set to (2, 0, 0) m/s and the parameter settings of the fluctuating component is presented in Table 2. The quadrotor is in hovering condition.
(3) Test 3: Time-varying wind estimation with a quadrotor in flight conditions. The time-varying wind field is set as that in Test 2. The quadrotor flies in the desired trajectory, and the real flight path is shown in Figure 6.

### 4.3. Simulation Results

**Figure 7:**
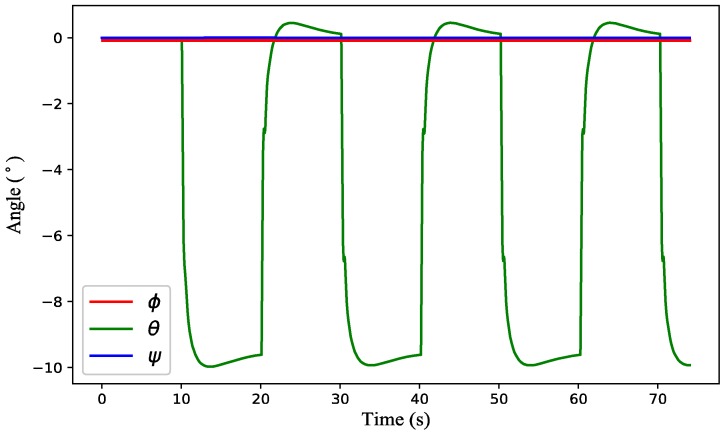
Attitude variation of the quadrotor when encountering gust.

**Figure 8:**
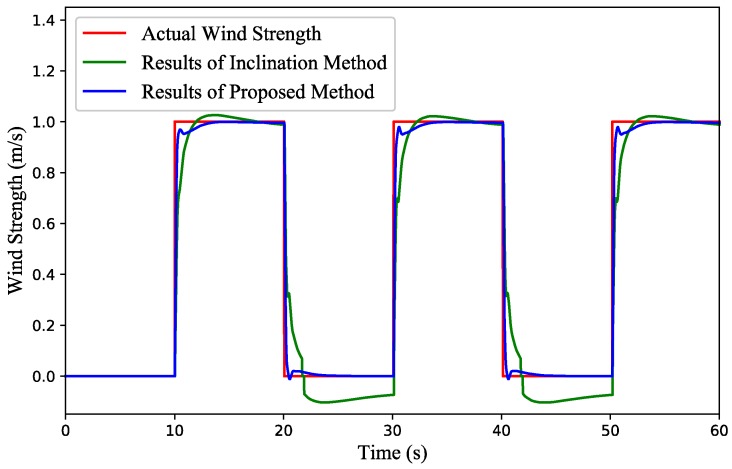
Simulation results of the gust wind estimation with a quadrotor in hovering condition.

**Figure 9:**
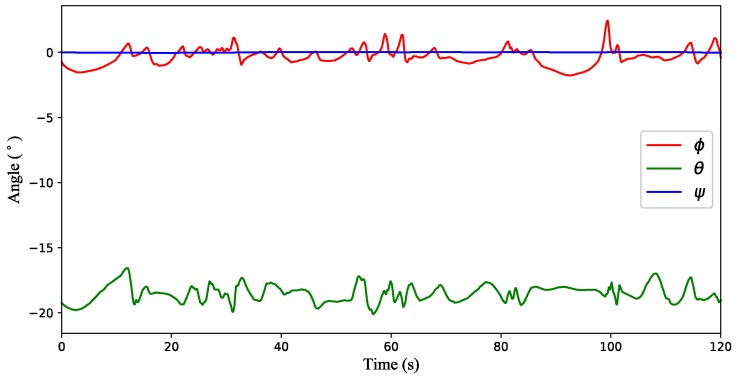
Attitude variation of the quadrotor hovering in the time-varying wind field.

**Figure 10:**
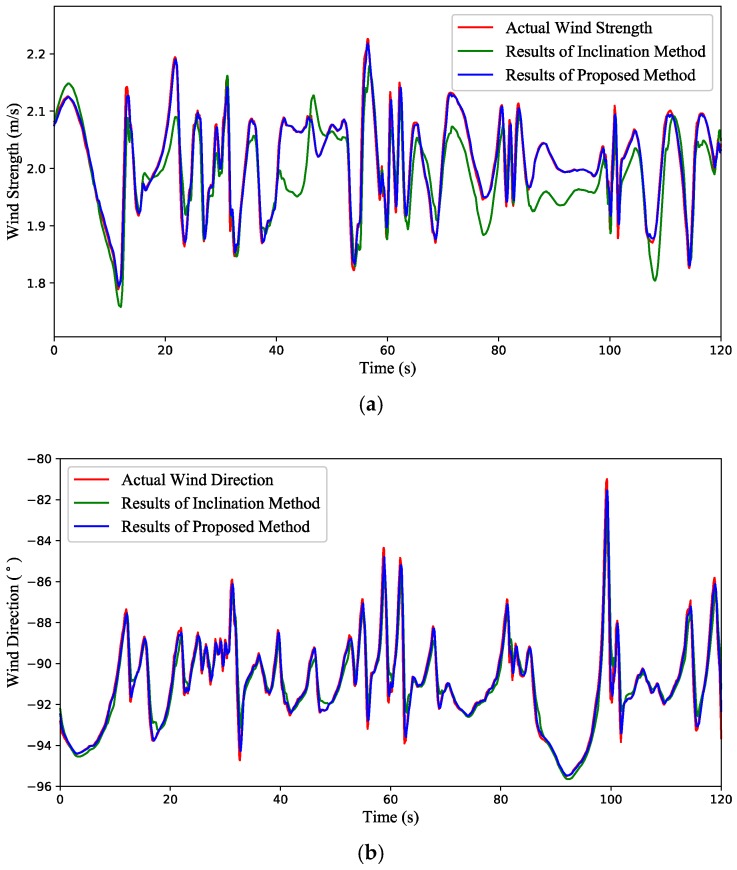
Simulation results of the time-varying wind estimation with a quadrotor in hovering condition. (**a**) The wind strength. (**b**) The wind direction.

**Figure 11:**
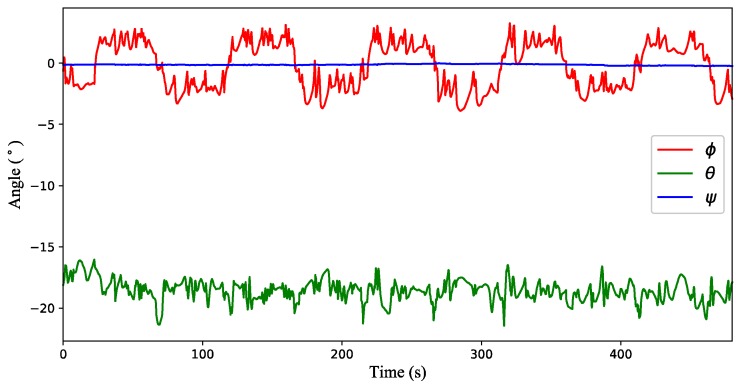
Attitude variation of the quadrotor flying in the time-varying wind field.

**Figure 12:**
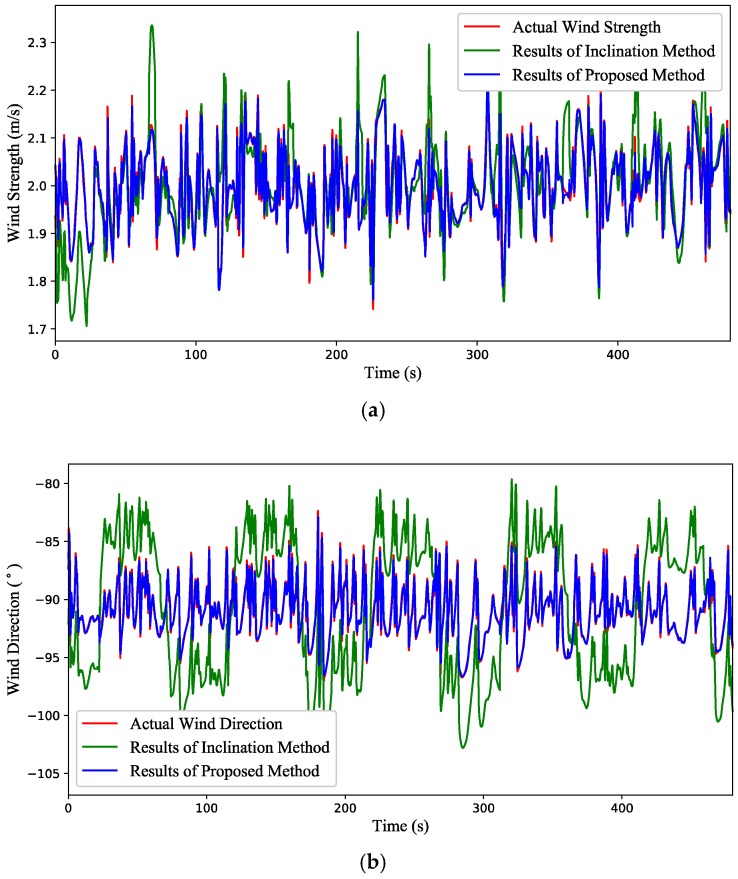
Simulation results of the time-varying wind estimation with a quadrotor in flight condition. (**a**) The wind strength. (**b**) The wind direction.

Figure 7, Figure 8, Figure 9, Figure 10, Figure 11 and Figure 12 illustrate the simulation results, and root mean squared errors (RMSEs) of the estimated wind velocities by two wind estimation methods are shown in Table 3.

(1) Test 1: Wind gust estimation with a quadrotor in hovering condition. The attitude variation of the quadrotor and simulation results are illustrated in Figure 7 and Figure 8, respectively. Note that the wind strength refers to the east-west wind strength component and the east is positive. It can be observed from Figure 8 that there always exist long-time negative outliers in the result by the inclination method when the wind suddenly stops. This is because when the wind stops suddenly, the quadrotor will move towards the previous upwind direction due to the attitude inertia. After detecting the position deviation, the controller will correct the attitude and pull the quadrotor back to the reference position. At this point, the quadrotor tends to tilt to the previous downwind direction, resulting in a negative false value. However, the result estimated by the proposed method is closer to the actual wind strength.

(2) Test 2: Time-varying wind estimation with a quadrotor in hovering condition. Figure 9 illustrates the attitude variation of the quadrotor, and simulation results are shown in Figure 10. It can be seen from Figure 10a that the wind strength estimated by the inclination method deviates from the true value in some time periods, while the result of the proposed method is almost coincident with the actual value. Since the north direction is set to 0°, the wind direction in Figure 10b fluctuates around −90 °. However, from the RMSE values in Table 3, the accuracy of the proposed method is better than that of the inclination method.

(3) Test 3: Time-varying wind estimation with a quadrotor in flight condition. The attitude variation of the quadrotor is shown in Figure 11. From the simulation results presented in Figure 12, there is a big deviation between the estimated value of the inclination method and the actual value for both wind strength and direction, while the estimated value of the proposed method is almost coincident with the actual one. When the quadrotor is in flight condition ($\dot{\xi }\neq \;0$), the inclination angle of the quadrotor not only contains the passive inclination caused by the air drag, but also includes the active inclination for flying along the target path. As a consequence, the inclination method will not work very well when the quadrotor is in flight condition.

## 5. Experiments and Results

Experimental tests for verifying the accuracy of wind estimation results by the quadrotor in hovering and flight conditions were carried out in an actual environment. The estimation results by the quadrotor and the measurement results by high-precision three-dimension (3D) anemometers were compared in the same wind environment. 

### 5.1. Experimental Setup

First of all, in order to calculate the thrust of the quadrotor, the quadratic sum of motors’ equivalent voltages with the hovering quadrotor was acquired in a windless environment. In a room with all windows and doors closed, the quadrotor automatically took off and stably hovered, and the measurement results of rotor motors’ PWM values and supply voltages were recorded for three minutes. The quadratic sum of the equivalent voltages remains stable and the mean value is 27.067.

The drag coefficient obtained by many groups of experiments using the method proposed in Section 3.4 is $\left[\begin{array}{ccc} 0.42 & 0.42 & 1.2 \end{array}\right]$, where *d* is set to 2 m and *h* is set to 1 m. Besides, other required parameters are the same as those of the simulation in Section 4.2.

**Figure 13:**
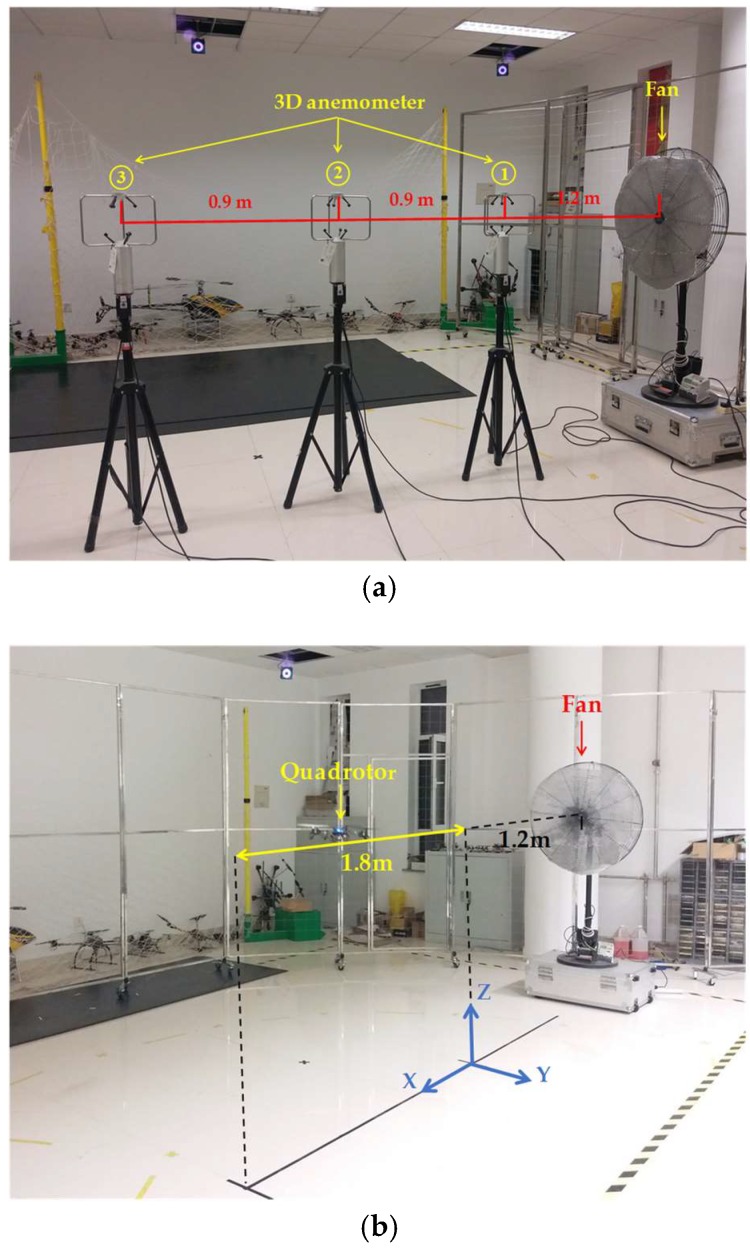
Scenes of the verification experiment. (**a**) Wind measurement by three 3D anemometers. (**b**) Wind estimation with a quadrotor.

The tests were carried out in an enclosed room and a certain intensity of wind toward a constant direction was generated by a modified industrial fan with a honeycomb. The scenarios of the verification experiments are illustrated in Figure 13. To ensure the uniformity and stability of the wind environment, the wind velocities in the straight line right to the center of the fan were measured by three 3D anemometers (Young 81000, R. M. Young Ltd, Traverse, MI, USA) arranged with a height of 1.4 m from the ground. The ultrasonic anemometer has a measurement resolution of 0.01 m/s for wind speed and 0.1° for wind direction, and its measurement ranges are 0–40 m/s and 0°–359.9° with the accuracies of ±1% and ± 2° (in the range of 0 to 30 m/s) for wind speed and direction, respectively. The sampling rate of the 3D anemometer is 30 Hz, and the wind velocity estimates were obtained at the same rate of 30 Hz.

The quadrotor was hovering at the location of Anemometer 2 in the verification experiment for hovering condition. For the test in flight condition, the quadrotor was controlled to make back-and-forth movements along the straight line between the locations of Anemometer 1 and Anemometer 3. Wind velocities were measured for 1 min and then the results were compared. 

### 5.2. Experimental Results

**Figure 14:**
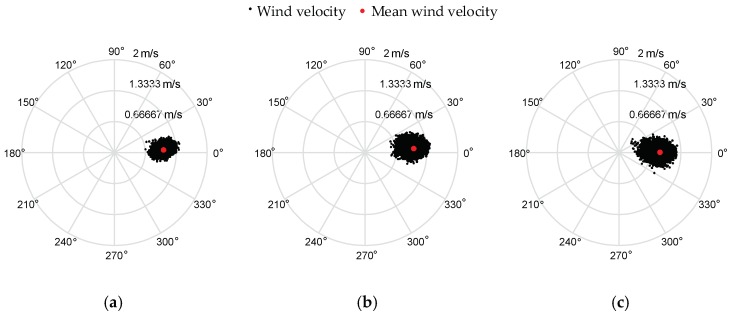
Measurement results by three 3D anemometers. (**a**–**c**) are the measurement results by Anemometers 1, 2 and 3, respectively.

**Figure 15:**
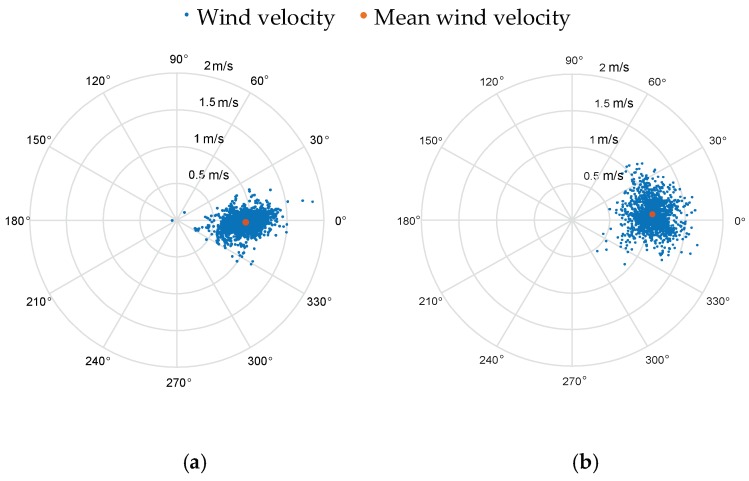

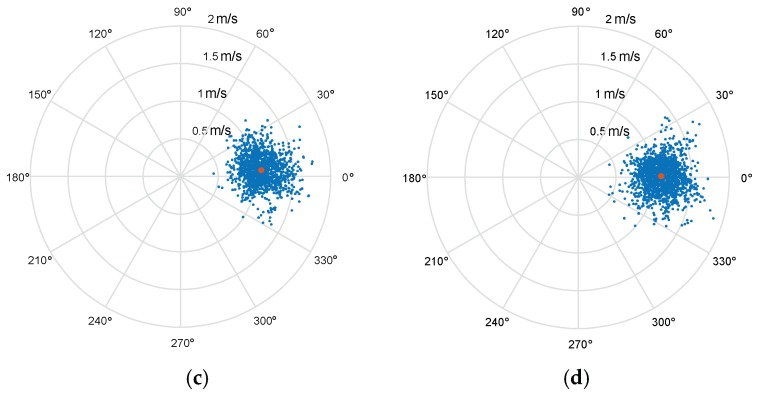
Estimation results by the quadrotor. (**a**) is the estimation result by the quadrotor in hovering condition. (**b**–**d**) are three sets of the estimation results by the quadrotor in flight condition.

The area of wind field generated by the fan is limited, and the wind along the centerline (see the yellow line in Figure 13b) of the fan is relatively stable, so the 3D anemometers were placed along the centerline. To compare the estimated results with the measured ones, the quadrotor should fly along the centerline. However, it may not fly exactly along the desired path, so the estimation results in the case that the quadrotor deviates too far from the established trajectory have been removed. The positons of the rotorcraft that meet the following equation were selected for comparison with the measured results:

(28) $$\sqrt{{\left(P_{y}-L_{y}\right)}^{2}+{\left(P_{z}-L_{z}\right)}^{2}}\leq 0.05\;m$$

where (*P_x_*, *P_y_*, *P_z_*) denotes the position of the quadrotor, and (*L_x_*, *L_y_*, *L_z_*) denotes the projections of the desired flight path on three axes, in which *L_y_* is 0 and *L_z_* is 1.36 m. The experimental results are plotted in the form of scatter diagram in polar coordinates in Figure 14 and Figure 15, and the red dot is the average value. The statistical properties of the measurement and estimation results are listed in Table 4 and Table 5, respectively. 

In the verification experiment for the quadrotor in hovering conditions, the quadrotor was hovering at the same location as Anemometer 2. Measurement results by Anemometer 2 and estimation results by the quadrotor are illustrated in Figure 14b and Figure 15a, respectively. The mean values of the estimated wind velocities are close to measurement results by Anemometer 2. It is observed from Table 4 and Table 5 that the mean wind speeds of the estimation results and the measurement results are both around 1 m/s, and the wind directions are close to zero. Besides, standard deviations of the estimation results are twice of the measurement ones.

In the verification experiment for the quadrotor in flight condition, three groups of flight tests by the quadrotor were conducted and the results are shown in Figure 15b–d. Although the anemometers are arranged with a certain distance, the wind strengths along the expected flight path of the quadrotor (i.e., the line between the locations of Anemometer 1 and Anemometer 3) are around 1 m/s, and the wind directions are close to zero. From the scatter diagrams in Figure 15b–d, the mean values of the estimated wind strengths and directions are very close to measurement results. However, the fluctuations of the estimation results are a little larger than the measurement results. The statistical properties show that the mean wind speeds of the estimation results are both around 1 m/s, and the wind directions are close to zero. The standard deviations of the estimation wind strengths and directions are about 2 and 3 times of the measurement results, respectively.

Given the above experimental results, besides the unevenness of the wind generated by the fan, the instability of the quadrotor also greatly increases the deviations of the estimation results. Since the fan is a small wind source which can only produce a limited range of wind field, when the quadrotor deviates from the ideal flight path, the estimated results are probably quite different from actual measurement data by the anemometers just towards the center of the fan. However, the measurement accuracy is sufficient to meet the requirement of wind data in odor tracking or other environmental monitoring tasks. In general, the wind velocity estimation method proposed in this paper can obtain reliable estimation values most of the time.

## 6. Conclusions

In this paper, we propose an ESO-based wind estimation method for unmanned rotorcrafts, which takes the flight acceleration into account. The parameters involved in this method are obtained by experiments performed in a windless environment, rather than designing a dedicated calibration device. As an instance, the quadrotor in both hovering and flight conditions demonstrates the performance of the proposed method in both simulations and experiments. The accuracy of the estimation results in actual environments can generally satisfy the requirements of the environment monitoring and gas tracing tasks. The verification experiments of the wind estimation method were conducted indoors and future work should focus on the wind estimation using real-time kinematic (RTK) GPS technology in outdoor tasks.

## Figures and Tables

**Table 1:** Reference values for the parameters in the model of the quadrotor.

Parameter	Value
*m* (kg)	0.122
*L* (m)	0.11
***J***	diag (0.0002632, 0.0002745, 0.00091175)
*k*	0.0000542
*b*	0.000011

**Table 2:** Reference values for the parameters in environmental wind.

Parameter	Value
$\mu_{x}{,\;\mu }_{y}{,\;\mu }_{z}$	0.3, 0.3, 0.3
$\zeta_{x}{,\;\zeta }_{y}{,\;\zeta }_{z}$	0.05, 0.05, 0.05
*G*	10
δ	∈[−0.5,0.5]

**Table 3:** RMSEs of the estimated wind velocities.

Simulation Scenario		RMSE of Wind Strength (m/s)	RMSE of Wind Direction (°)
Test 1	Inclination method	0.1287	/
	Proposed method	0.0796	/
Test 2	Inclination method	0.0481	0.6889
	Proposed method	0.0154	0.3723
Test 3	Inclination method	0.0708	5.4621
	Proposed method	0.0156	0.4558

**Table 4:** Measurement results of the anemometers.

Statistical Indicators	Anemometer 1	Anemometer 2	Anemometer 3
Mean value of wind strength (m/s)	1.0616	1.0551	0.8812
Mean value of wind direction (°)	3.1240	4.6850	0.5207
Standard deviation of wind strength (m/s)	0.0941	0.1088	0.1208
Standard deviation of wind direction (°)	3.1985	4.3714	5.3590

**Table 5:** Estimation results of the quadrotor.

Statistical Indicators	Hover Test	Flight Test 1	Flight Test 2	Flight Test 3
Mean value of wind strength (m/s)	0.9384	1.0681	1.0773	1.0983
Mean value of wind direction (°)	−1.7664	4.2946	4.4181	0.8479
Standard deviation of wind strength (m/s)	0.1751	0.1710	0.1910	0.2009
Standard deviation of wind direction (°)	9.3774	11.2742	10.6558	10.6584
